# Effect of an Individually Tailored and Home-Based Intervention in the Chronic Phase of Traumatic Brain Injury: A Randomized Clinical Trial

**DOI:** 10.1001/jamanetworkopen.2023.10821

**Published:** 2023-05-05

**Authors:** Ida M. H. Borgen, Marianne Løvstad, Solveig L. Hauger, Marit V. Forslund, Ingerid Kleffelgård, Nada Andelic, Unni Sveen, Helene L. Søberg, Solrun Sigurdardottir, Laraine Winter, Marte Ørud Lindstad, Cathrine Brunborg, Cecilie Røe

**Affiliations:** 1 Department of Physical Medicine and Rehabilitation, Oslo University Hospital, Oslo, Norway; 2 Department of Psychology, Faculty of Social Sciences, University of Oslo, Oslo, Norway; 3 Department of Research, Sunnaas Rehabilitation Hospital, Nesoddtangen, Norway; 4 Center for Habilitation and Rehabilitation Models and Services, Institute of Health and Society, Faculty of Medicine, University of Oslo, Oslo, Norway; 5 Department for Occupational Therapy, Prosthetics and Orthotics, Faculty of Health Sciences, Oslo Metropolitan University, Oslo, Norway; 6 Department of Physiotherapy, Faculty of Health Sciences, Oslo Metropolitan University, Oslo, Norway; 7 Centre for Rare Disorders, Oslo University Hospital, Oslo, Norway; 8 M. Louise Fitzpatrick College of Nursing, Villanova University, Villanova, Pennsylvania; 9 Department of Health Sciences in Gjøvik, Faculty of Medicine and Health Sciences, Norwegian University of Science and Technology, Gjøvik, Norway; 10 Oslo Centre for Biostatistics and Epidemiology, Oslo University Hospital, Oslo, Norway; 11 Institute of Clinical Medicine, Faculty of Medicine, University of Oslo, Oslo, Norway

## Abstract

**Importance:**

Traumatic brain injury (TBI) can cause long-lasting and heterogeneous difficulties that require an individually tailored approach to rehabilitation. However, high-quality studies of treatment options in the chronic phase of TBI are lacking.

**Objective:**

To evaluate the effect of a home-based, individualized, and goal-oriented rehabilitation intervention in the chronic phase of TBI.

**Design, Setting, and Participants:**

This study was an intention-to-treat parallel-group assessor-blinded randomized clinical trial with 1:1 randomization to an intervention or control group. Participants included adults in southeastern Norway who had sustained a TBI more than 2 years earlier, lived at home, and had ongoing TBI-related difficulties. A population-based sample of 555 individuals were invited, and 120 were included. Participants were assessed at baseline, 4 months, and 12 months after inclusion. Specialized rehabilitation therapists provided the intervention in patients’ homes or via video conference and telephone. Data collection was conducted between June 5, 2018, and December 14, 2021.

**Interventions:**

The intervention group received an 8-session individually tailored and goal-oriented rehabilitation program over 4 months. The control group received usual care in their municipality.

**Main Outcomes and Measures:**

Preestablished primary outcomes were disease-specific health-related quality of life (HRQOL; measured by the Quality of Life After Brain Injury [QOLIBRI] overall scale) and social participation (measured by the Participation Assessment With Recombined Tools–Objective [PART-O] social subscale). Preestablished secondary outcomes included generic HRQOL (measured by the EuroQol 5-dimension 5-level [EQ-5D-5L] questionnaire), difficulty with TBI-related problem management (target outcomes; mean severity calculated across 3 main self-identified problem areas that were individually measured using a 4-point Likert scale), TBI symptoms (measured by the Rivermead Post Concussion Symptoms Questionnaire [RPQ]), psychological distress (depression and anxiety; measured by the Patient Health Questionnaire 9-item scale and the Generalized Anxiety Disorder 7-item scale [GAD-7], respectively), and functional competency (measured by the Patient Competency Rating Scale).

**Results:**

Among 120 participants in the chronic phase of TBI, the median (IQR) age was 47.5 (31.0-55.8) years, and the median (IQR) time since injury was 4 (3-6) years; 85 (70.8%) were male. A total of 60 participants were randomized to the intervention group, and 60 were randomized to the control group. Between baseline and 12 months, no significant between-group effects were found for the primary outcomes of disease-specific HRQOL (QOLIBRI overall scale score: 2.82; 97.5% CI, −3.23 to 8.88; *P* = .30) or social participation (PART-O social subscale score: 0.12; 97.5% CI, −0.14 to 0.38; *P* = .29). At 12 months, the intervention group (n = 57) had significantly higher generic HRQOL (EQ-5D-5L score: 0.05; 95% CI, 0.002-0.10; *P* = .04) and fewer symptoms of TBI (RPQ total score: −3.54; 95% CI, −6.94 to −0.14; *P* = .04) and anxiety (GAD-7 score: −1.39; 95% CI, −2.60 to −0.19; *P* = .02) compared with the control group (n = 55). At 4 months only, the intervention group (n = 59) had significantly less difficulty managing TBI-related problems (target outcomes mean severity score: −0.46, 95% CI, −0.76 to −0.15; *P* = .003) compared with the control group (n = 59). No adverse events were reported.

**Conclusions and Relevance:**

In this study, no significant results were observed for the primary outcomes of disease-specific HRQOL or social participation. However, the intervention group reported improvements in secondary outcomes (generic HRQOL and symptoms of TBI and anxiety) that were maintained at 12-month follow-up. These findings suggest that rehabilitation interventions could help patients even in the chronic phase of TBI.

**Trial Registration:**

ClinicalTrials.gov Identifier: NCT03545594

## Key Points

**Question:**

Does a home-based intervention that is individually tailored and goal oriented improve health-related quality of life (HRQOL) and participation and ameliorate symptoms in the chronic phase of traumatic brain injury (TBI)?

**Findings:**

In this randomized clinical trial including 120 adult participants in the chronic phase of TBI, no significant results were seen in disease-specific HRQOL or social participation. However, generic HRQOL and TBI- and anxiety-related symptom levels improved significantly in the intervention group compared with the control group.

**Meaning:**

These findings suggest that an individually tailored and goal-oriented rehabilitation program might be effective in improving generic HRQOL and symptom burden in the chronic phase of TBI.

## Teaser

This randomized clinical trial assesses the effect of a home-based, individualized, and goal-oriented rehabilitation intervention among adult patients with chronic traumatic brain injury.

## Introduction

Traumatic brain injury (TBI) is a chronic disease process with potential lifelong impact on health and well-being.^1,2^ Reductions in health-related quality of life (HRQOL) and participation are commonly reported.^3,4,5^ Despite extensive knowledge about common TBI-related sequelae, research has consistently documented worldwide unmet needs for health care services in the chronic phase of TBI,^6,7^ even in affluent countries with universal health care services.^8^

There are few methodologically rigorous studies that document treatment options in the chronic phase of TBI. The World Health Organization Rehabilitation 2030 Initiative^9^ suggests the need for high-quality studies that document the efficacy of rehabilitation interventions. However, some studies^10,11^ have found that rehabilitation can improve QOL and community integration among patients in the chronic phase of TBI. The Cognitive Rehabilitation Task Force of the American Congress of Rehabilitation Medicine^12^ recommends as a practice standard the provision of holistic neuropsychological rehabilitation targeting cognitive, emotional, and interpersonal difficulties in patients with chronic consequences of acquired brain injury. Furthermore, bringing interventions to the patient’s home environment might be especially important.^13^ However, a systematic review^14^ found that studies of community-based rehabilitation in the chronic phase of acquired brain injury are characterized by small samples, unclear description of intervention content, and lack of common outcome measures.

A challenge when investigating interventions among patients with TBI is the highly heterogeneous consequences of the injury. Interventions should further be aimed not only at symptom reduction, but also at improving HRQOL and participation. To better meet the challenges of individual patients, TBI interventions should be tailored to the specific problem profiles reported by the patient rather than being prespecified by researchers.^15^ Goal-oriented rehabilitation is an approach that has been found to increase the patient-centeredness of interventions as well as patient self-efficacy and motivation.^16^ Overall, individually tailored and goal-oriented interventions might address unmet rehabilitation needs in patients living with the chronic consequences of TBI, but these interventions need more investigation.

A randomized clinical trial (RCT) of a home-based goal-oriented intervention has been conducted in the US by Winter et al.^13^ Their study included 81 veterans and their family members; at the end of intervention, the intervention group had less difficulty managing self-identified TBI-related problems (ie, target outcomes) and higher levels of community integration than the control group. However, this trial^13^ did not include long-term follow-up, and the intervention needs to be replicated in a civilian population with more severe injuries. In the current study, the intervention manual used by Winter et al^13^ was adapted to investigate the effect of the intervention among civilian patients with TBI of all severities in the context of a health care system with universal access. The objective of this RCT was to establish whether a home-based, goal-oriented, and individualized rehabilitation program would be effective in improving HRQOL, social participation, and target outcomes and in reducing TBI-related and psychiatric symptoms.

## Methods

This study followed the Consolidated Standards of Reporting Trials (CONSORT) guideline for RCTs.^17^ The trial was approved by the Data Protection Office at Oslo University Hospital. All participants provided written informed consent.

### Trial Design

This trial was a parallel 2-group RCT with 1:1 randomization to either an intervention or control group. Participants were assessed at baseline before randomization. Outcome assessments were performed at 4 months after inclusion (after the end of intervention, some assessments were performed between 4 and 5 months) and again at 12 months after inclusion. Full details are provided in the trial protocol in Supplement 1.

### Participants

The principal investigator (C.R.) identified potentially eligible participants by screening patients in southeastern Norway who were included in previous studies and outpatient registries, who then were invited by letter. Patients were thereafter contacted by telephone and screened for eligibility. Inclusion criteria included a diagnosis of TBI (based on *International Classification of Diseases, Tenth Revision, Clinical Modification* codes S06.01-S06.09) with radiologically verified intracranial abnormalities; age 18 to 72 years; time since injury of more than 2 years; living at home, and presence of ongoing TBI-related difficulties. Exclusion criteria included inability to provide informed consent or collaborate in the goal-setting process, presence of a severe ongoing neurological or psychiatric condition, and/or active misuse of substances. Due to the COVID-19 pandemic, the intervention was delivered mainly by video conference for participants recruited after March 12, 2020. Ability to use a computer was therefore added as an inclusion criterion. Participants were encouraged to include a family member or close friend as a coparticipant if available. Effort was made to include a sample representative of patients seen in our outpatient clinic (eg, not excluding patients with psychiatric disorders, except those with severe ongoing psychiatric conditions, such as psychosis). The full eligibility criteria are provided in the Box. If eligible, participants performed the baseline assessment at Oslo University Hospital. Recruitment was conducted between June 5, 2018, and December 14, 2020, stopping when the predefined sample size was reached. A population-based sample of 555 individuals were invited to participate, and 120 were included.

Box. **Eligibility Criteria**

**Inclusion Criteria**

- TBI diagnosis (based on *ICD-10-CM* codes S06.01-S06.09); radiologically verified intracranial abnormalities
- Age 18-72 y; age ≥16 y at time of injury
- Time since injury ≥2 y
- Lives at home
- Ongoing TBI-related cognitive, emotional, and/or physical problems and/or reduced physical and mental health and/or difficulties with participation in activities with family, friends, and/or in the community
- Able to use computer or tablet computer; internet access (added criterion because of the COVID-19 pandemic)

**Exclusion Criteria**

- Unable to provide informed consent
- Severe progressive neurological condition or severe ongoing psychiatric disorder that may confound outcomes
- Unable to collaborate in goal-setting process
- Insufficient command of Norwegian language (cannot communicate with rehabilitation therapists or respond to questionnaires)
- Active substance misuse and/or violent tendencies that may put rehabilitation therapists at risk

Abbreviations: *ICD-10-CM*, *International Classification of Diseases, Tenth Revision, Clinical Modification*; TBI, traumatic brain injury.

Demographic and injury-related data were collected; data on race and ethnicity were not collected because it is not conventional in Norwegian research practice. All participants were evaluated using the Glasgow Outcome Scale–Extended version (score range, 1-8, with 1 indicating death and 7-8 indicating good recovery).^18^ If eligibility was confirmed at baseline and written consent provided, participants were randomized to the intervention or control group. The intervention was delivered in the participant’s home or by video conference or telephone. Assessments at 4 months and 12 months after inclusion were conducted either at the hospital or through telephone interviews and mailed questionnaires. All participants were asked about the services they were receiving at all time points using the Needs and Provisions Complexity Scale–Gets subscale, which measures levels of service provided (score range, 0 to 25, with higher scores indicating higher levels of service).^19^ Data were collected between June 5, 2018, and December 14, 2021.

### Intervention Group

The intervention group received an 8-session rehabilitation program over 4 months. In-home and video conference sessions typically lasted 2 hours, while telephone sessions typically lasted 1 hour. Four experienced rehabilitation therapists (psychologist [I.M.H.B.], neuropsychologist [S.L.H.], physician [M.V.F.], and physiotherapist [I.K.]) delivered the intervention, and 1 rehabilitation therapist (I.M.H.B., S.L.H., M.V.F., or I.K.) followed up with each participant throughout the intervention. If available and relevant, family members or local health care personnel participated in sessions.

An overview of intervention content is available in the eFigure in Supplement 2. At baseline, participants identified 3 main problem areas (target outcomes). In the first sessions, participants were asked whether they wished to start working on any of these problems or another TBI-related difficulty. When a problem area was chosen, the rehabilitation therapist then guided the participant to brainstorm about the nature of the problem and the changes the participant wished to achieve. A SMART goal approach was used, in which the participant was encouraged to set goals that were specific, measurable, achievable, realistic or relevant, and timed. For each goal, goal attainment scaling^20^ was performed to quantify baseline levels, the expected level, and actual goal attainment. Rehabilitation strategies for goal attainment were then established in an action plan. Strategies were based on suggestions made by participants, family members, and rehabilitation therapists and mainly involved environmental support and compensatory strategies. Suggestions from rehabilitation therapists were informed by knowledge about evidence-based treatment approaches related to the functional domain in question. The rehabilitation therapists kept up to date through literature searches and had clinical discussions in cases of new issues. In addition, 3 psychoeducational topics (cognitive impairment after TBI, stress management and mindfulness, and cognitive communication difficulties) were discussed with all participants. Treatment fidelity was ensured both by calibration during a feasibility study^21^ as well as frequent meetings among rehabilitation therapists and evaluation of the rehabilitation therapists’ adherence to the intervention manual (as adapted from the manual by Winter et al^13^) by senior researchers (M.L., S.L.H., I.K., and C.R.) in 10% of sessions.

### Control Group

All control group participants received feedback on their baseline assessment, and a brief report was sent to their general practitioner. Because an active control group was not feasible, the control group continued to receive any concomitant care (registered at each time point) they were already receiving, with no additional treatment. In Norway, municipal health care services are mainly responsible for treatment in the chronic phase of TBI. There are no specialized TBI services in the communities, but specialized health care services at hospitals are provided when needed (eg, in cases of moderate to severe psychiatric disorders or epilepsy).

### Outcomes

Two primary outcomes, disease-specific HRQOL and participation, were defined based on experiences from the feasibility study.^21^ Disease-specific HRQOL was measured using the Quality of Life After Brain Injury (QOLIBRI) overall scale (score range, 0-100, with higher scores indicating better HRQOL).^22^ Participation was measured using the Participation Assessment With Recombined Tools–Objective (PART-O) social subscale (score range, 0-5, with higher scores indicating higher levels of participation).^23,24^ Because the COVID-19 pandemic influenced participation options, the PART-O may not have been the ideal outcome measure. The social subscale was believed to be the least influenced by COVID-19 restrictions and was selected as the index of participation before statistical analyses.

Secondary outcomes included target outcomes, generic HRQOL, TBI symptoms, depression- and anxiety-related symptoms, and functional competency. Outcome measures consisted of 1 interview-based assessment and 5 questionnaires. For the target outcomes, an interview-based measure was adapted from the study by Winter et al.^13^ At baseline, participants were asked to identify 3 main ongoing problems related to their TBI and the current degree of difficulty in managing each of the problems on a Likert scale from 0 to 4 (with 0 indicating not difficult at all and 4 indicating extremely difficult). Rehabilitation therapists would prompt participants to identify problem areas related to activities in their everyday lives to ensure they would be amenable to intervention. A mean severity score across the 3 target outcomes was calculated. For the other secondary outcomes, 5 questionnaires were used: the EuroQol 5-dimension 5-level questionnaire (EQ-5D-5L; score range, 0-1, with higher scores indicating better HRQOL)^25^ to measure generic HRQOL, the Rivermead Post Concussion Symptoms Questionnaire (RPQ; score range, 0-64, with higher scores indicating greater severity of symptoms)^26^ to measure TBI symptoms, the Patient Health Questionnaire 9-item scale (score range, 0-27, with higher scores indicating greater severity of depression)^27^ and the Generalized Anxiety Disorder 7-item scale (GAD-7; score range, 0-21, with higher scores indicating greater severity of anxiety)^28^ to measure depression- and anxiety-related symptoms, and the patient version of the Patient Competency Rating Scale (score range, 30-150, with higher scores indicating greater competency)^29^ to assess functional competency.

### Sample Size

Sample size calculations were conducted before the trial and were based on between-group differences in the change in primary outcomes. The significance level was Bonferroni corrected for a clinically meaningful between-group mean difference of 12% on the QOLIBRI overall scale (with an estimated SD of 20%) and a between-group mean difference of 1.8 on the PART-O (with an estimated SD of 3.0). Thus, with equal randomization to treatment groups, power of 80%, and a significance level of α = .025 (correcting for 2 primary outcomes), the sample size was calculated as 55 patients in each group for both primary outcomes. Allowing for an attrition rate of 10%, 60 participants were included in each treatment group. Sample size calculations were performed using G*Power software, version 3 (Faul et al^30^).

### Randomization

Participants were randomized 1:1 by web-based block allocation (variable sizes of 4 and 6) generated in Stata software, version 15 (StataCorp LLC), by an independent statistician, and the randomization list was provided in a fixed sequential order. The allocation sequence could only be accessed by the principal investigator (C.R.), who was not involved in inclusion procedures. Rehabilitation therapists were responsible for the inclusion of participants and sent a sham number to the principal investigator via email to receive information on group allocation.

### Blinding

Outcome assessors were blinded. Statistical analyses were conducted by an independent statistician. The first author (I.M.H.B.) wrote the Results section of this article while blinded to group allocation.

### Statistical Analysis

Linear mixed-effects models were fitted to primary and secondary outcome variables with time and time-by-treatment interaction as categorical fixed effects. The main effect of treatment was removed from the model to adjust for potential baseline differences in the outcome. The models included a random intercept and a random effect for time. Three variables (PART-O social subscale score, QOLIBRI overall scale score, and GAD-7 score) could not be modeled with the random effect for time; thus, only a random intercept was used. Based on the linear mixed-effects models, mean values for primary outcomes were estimated with 97.5% CIs for all time points (baseline, 4 months, and 12 months) for each group. We also estimated mean within-group and between-group differences in change from baseline to 4 months and from baseline to 12 months. Analyses were performed using the intention-to-treat approach. To accommodate for having 2 independent primary outcomes, a conservative significance level of 2-tailed *P* < .025 was applied. For secondary outcomes, mean values with 95% CIs were estimated, and a significance level of 2-tailed *P* < .05 was applied. Statistical analyses were performed using Stata software, version 17.

## Results

**Figure 1.**
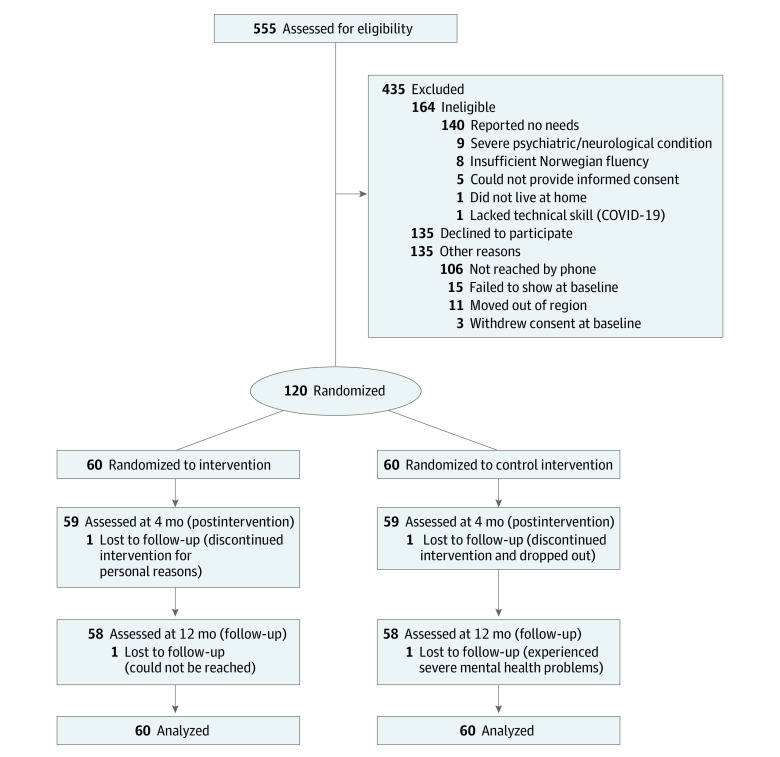
Study Flowchart

Among 120 participants included in the intention-to-treat analysis, the median (IQR) age was 47.5 (31.0-55.8) years, and the median (IQR) time since injury was 4 (3-6) years; 85 participants (70.8%) were male and 35 (29.2%) were female. A total of 60 participants were randomized to the intervention group (median [IQR] age, 45.5 [29.5-54.0] years; 44 men [73.1%]) and 60 to the control group (median [IQR] age, 49.0 [33.0-60.5] years; 41 men [68.3%]). Overall, 4 participants withdrew from the study or could not be reached at 12 months. The study flowchart is shown in Figure 1.

Baseline demographic and clinical characteristics were generally similar between groups (Table 1). A total of 73 participants (60.8%) had a participating family member, of whom 55 (75.3%) were spouses. Any concurrent services (both TBI- and non–TBI-related) provided in specialized and municipal care were also similar between the intervention and control groups at baseline, 4 months, and 12 months, as was the rate of participants who were married or in domestic partnerships at all time points. No adverse or unintended effects were reported.

**Table 1.** Demographic and Clinical Characteristics of the Intervention and Control Groups

Characteristic	Participants, No. (%)	
	Intervention group (n = 60)	Control group (n = 60)
Age, median (IQR), y	45.5 (29.5-54.0)	49.0 (33.0-60.5)
Sex		
Male	44 (73.3)	41 (68.3)
Female	16 (26.7)	19 (31.7)
Educational level, mean (SD), y	13.2 (2.3)	13.1 (2.3)
Paid employment	31 (51.7)	29 (48.3)
Married or domestic partner	32 (53.3)	36 (60.0)
No. of self-reported comorbid conditions, median (IQR)	2 (1-3)	2 (1-3)
Months after injury, median (IQR)	52.0 (44.0-83.0)	53.5 (44.0-80.0)
Lowest unsedated GCS score within first 24 h, median (IQR)	8 (4-14)^a^	10 (6-14)^b^
TBI severity^c^		
Mild	16 (26.7)	25 (41.7)
Moderate	10 (16.7)	8 (13.3)
Severe	30 (50.0)	24 (40.0)
Unknown	4 (6.7)	3 (5.0)
Cause of injury		
Transportation-related accident	24 (40.0)	26 (43.3)
Fall	17 (28.3)	22 (36.7)
Violent incident	4 (6.7)	5 (8.3)
Other	12 (20.0)	6 (10.0)
Unknown	3 (5.0)	1 (1.7)
GOS-E score, median (IQR)^d^	6 (5-6)	6 (5-7)
Receipt of services at baseline (NPCS-Gets)		
No follow-up	18 (30.0)	17 (28.3)
Municipal services	9 (15.0)	14 (23.3)
Specialized health care services	15 (25.0)	15 (25.0)
Municipal services and specialized health care services	18 (30.0)	14 (23.3)

Abbreviations: GCS, Glasgow Coma Scale; GOS-E, Glasgow Outcome Scale–Extended; NPCS-Gets, Needs and Provisions Complexity Scale–Gets subscale (measuring levels of service provided); TBI, traumatic brain injury.

^a^ Among 56 patients.

^b^ Among 57 patients.

^c^ Severity was measured using the GCS, with scores of 13 to 15 indicating mild TBI, 9 to 12 indicating moderate TBI, and 3 to 8 indicating severe TBI.

^d^ Score range, 1 to 8, with 1 indicating death and 7 to 8 indicating good recovery.

**Figure 2.**
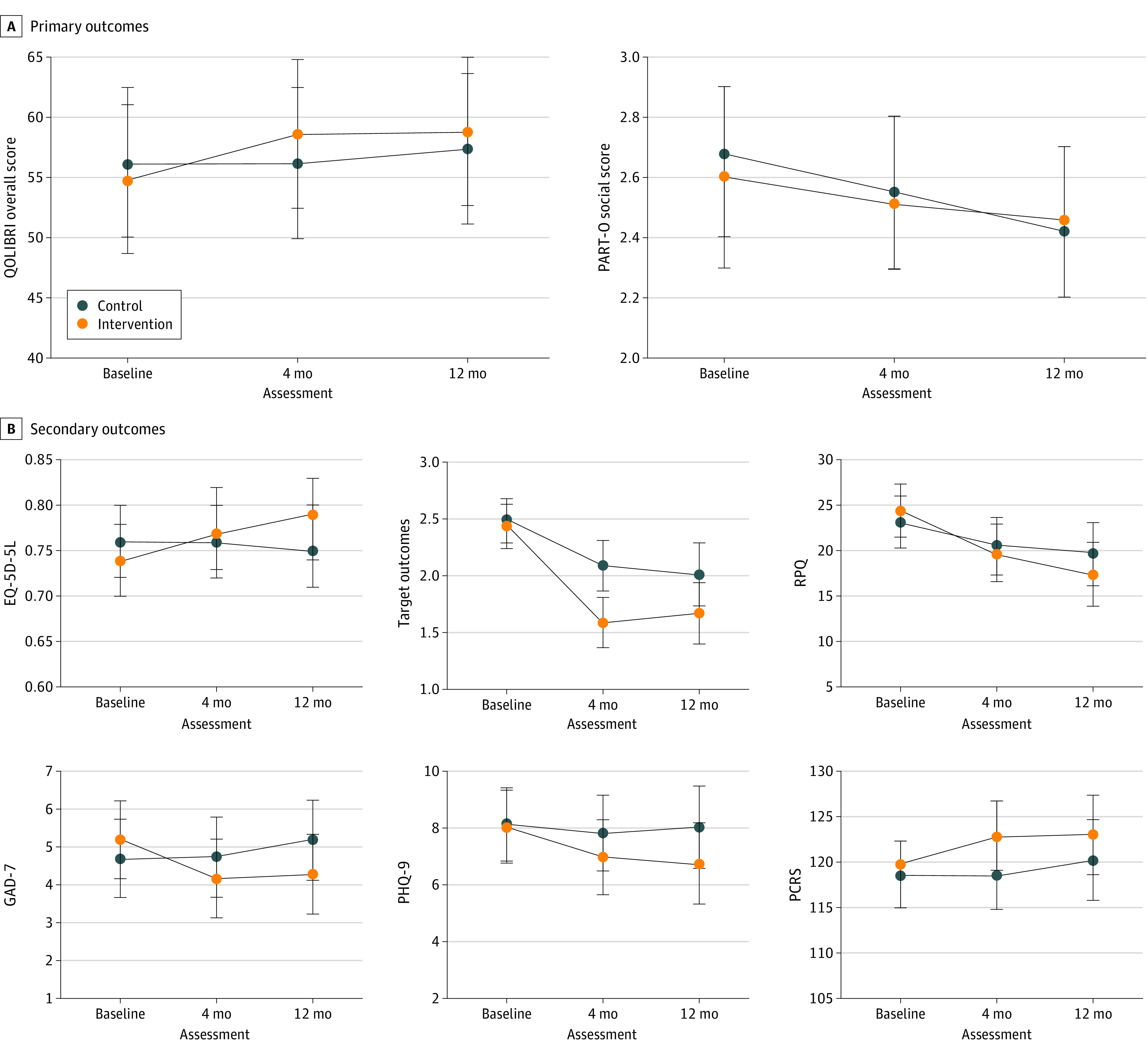
Linear Mixed Model Results for Primary and Secondary Outcomes EQ-5D-5L indicates EuroQol 5-dimension 5-level; GAD-7, Generalized Anxiety Disorder 7-item scale; PART-O, Participation and Recombined Tools–Objective social participation subscale; PCRS, Patient Competency Rating Scale; PHQ-9, Patient Health Questionnaire 9-item scale; QOLIBRI, Quality of Life After Brain Injury; and RPQ, Rivermead Post Concussion Symptoms Questionnaire.

From baseline to 12 months, no statistically significant between-group differences were found for the primary outcome measures of HRQOL (QOLIBRI overall scale score: 2.82; 97.5% CI, −3.23 to 8.88; *P* = .30) or social participation (PART-O social subscale score: 0.12; 97.5% CI, −0.14 to 0.38; *P* = .29). For the secondary outcomes, a statistically significant difference at 12 months was observed in favor of the intervention group (n = 57) vs the control group (n = 55) for generic HRQOL (EQ-5D-5L score: 0.05; 95% CI, 0.002-0.10; *P* = .04), TBI symptoms (RPQ total score: −3.54; 95% CI, −6.94 to −0.14; *P* = .04), and anxiety symptoms (GAD-7 total score: −1.39; 95% CI, −2.60 to −0.19; *P* = .02). At 4 months only, participants in the intervention group (n = 59) had significantly less difficulty managing main TBI-related problems (target outcomes mean severity score: −0.46; 95% CI, −0.76 to −0.15; *P* = .003) compared with participants in the control group (n = 59). Full results of the linear mixed model are shown in Table 2 and Figure 2.

**Table 2.** Linear Mixed-Effects Model Results for Primary and Secondary Outcomes

Outcome	Intervention group (n = 60)				Control group (n = 60)				Between-group change from baseline	
	No. of patients	Mean (97.5% or 95% CI)^a^	Change from baseline, mean (97.5% or 95% CI)^a^	*P* value	No. of patients	Mean (97.5% or 95% CI)^a^	Change from baseline, mean (97.5% or 95% CI)^a^	*P* value	Mean (97.5% or 95% CI)^a^	*P* value
**Primary**										
QOLIBRI overall scale score^b^										
Baseline	60	54.86 (48.75 to 60.97)	NA	NA	60	56.25 (50.14 to 62.36)	NA	NA	NA	NA
4 mo	58	58.58 (52.43 to 64.73)	3.72 (−0.51 to 7.95)	.049	55	56.19 (49.99 to 62.40)	−0.06 (−4.36 to 4.25)	.98	3.77 (−2.26 to 9.81)	.16
12 mo	57	58.83 (52.66 to 65.00)	3.97 (−0.29 to 8.23)	.04	55	57.40 (51.19 to 63.60)	1.15 (−3.16 to 5.45)	.55	2.82 (−3.23 to 8.88)	.30
PART-O social subscale score^c^										
Baseline	60	2.60 (2.34 to 2.85)	NA	NA	60	2.67 (2.42 to 2.93)	NA	NA	NA	NA
4 mo	58	2.51 (2.26 to 2.77)	−0.08 (−0.27 to 0.10)	.29	55	2.54 (2.28 to 2.80)	−0.13 (−0.32 to 0.05)	.10	0.05 (−0.21 to 0.31)	.67
12 mo	57	2.46 (2.21 to 2.72)	−0.14 (−0.32 to 0.05)	.09	55	2.42 (2.16 to 2.67)	−0.26 (−0.44 to −0.07)	.002	0.12 (−0.14 to 0.38)	.29
**Secondary**										
Target outcomes mean severity score^d^										
Baseline	60	2.44 (2.24 to 2.63)	NA	NA	60	2.49 (2.29 to 2.68)	NA	NA	NA	NA
4 mo	59	1.58 (1.36 to 1.81)	−0.86 (−1.07 to −0.64)	<.001	59	2.09 (1.86 to 2.31)	−0.40 (−0.62 to −0.18)	<.001	−0.46 (−0.76 to −0.15)	.003
12 mo	58	1.67 (1.39 to 1.94)	−0.77 (−1.01 to −0.53)	<.001	58	2.01 (1.73 to 2.29)	−0.48 (−0.72 to −0.24)	<.001	−0.29 (−0.63 to 0.05)	.09
EQ-5D-5L score^e^										
Baseline	60	0.74 (0.70 to 0.78)	NA	NA	60	0.76 (0.72 to 0.80)	NA	NA	NA	NA
4 mo	58	0.77 (0.73 to 0.82)	0.04 (0.01 to 0.07)	.009	55	0.76 (0.72 to 0.80)	0.01 (−0.02 to 0.04)	.62	0.03 (−0.01 to 0.07)	.14
12 mo	57	0.79 (0.74 to 0.83)	0.05 (0.01 to 0.08)	.007	55	0.75 (0.71 to 0.80)	−0.003 (−0.04 to 0.03)	.87	0.05 (0.002 to 0.10)	.04
RPQ total score^f^										
Baseline	60	24.40 (21.47 to 27.33)	NA	NA	60	23.12 (20.18 to 26.05)	NA	NA	NA	NA
4 mo	58	19.73 (16.57 to 22.88)	−4.67 (−6.95 to −2.39)	<.001	55	20.52 (17.34 to 23.70)	−2.60 (−4.92 to −0.28)	.03	−2.08 (−5.33 to 1.18)	.21
12 mo	57	17.37 (13.90 to 20.83)	−7.03 (−9.42 to −4.64)	<.001	55	19.62 (16.14 to 23.11)	−3.49 (−5.91 to −1.07)	.005	−3.54 (−6.94 to −0.14)	.04
PHQ-9 total score^g^										
Baseline	60	8.02 (6.74 to 9.30)	NA	NA	60	8.08 (6.80 to 9.36)	NA	NA	NA	NA
4 mo	58	6.96 (5.64 to 8.27)	−1.06 (−1.96 to −0.16)	.02	55	7.81 (6.49 to 9.13)	−0.27 (−1.19 to 0.64)	.56	−0.78 (−2.06 to 0.50)	.23
12 mo	57	6.73 (5.31 to 8.16)	−1.28 (−2.34 to −0.22)	.02	55	8.03 (6.59 to 9.47)	−0.05 (−1.12 to 1.01)	.92	−1.23 (−2.73 to 0.27)	.11
GAD-7 total score^h^										
Baseline	60	5.18 (4.15 to 6.22)	NA	NA	60	4.70 (3.67 to 5.73)	NA	NA	NA	NA
4 mo	58	4.18 (3.13 to 5.22)	−1.01 (−1.85 to −0.17)	.02	55	4.75 (3.69 to 5.80)	0.05 (−0.81 to 0.90)	.92	−1.05 (−2.25 to 0.15)	.09
12 mo	57	4.28 (3.23 to 5.32)	−0.91 (−1.75 to −0.06)	.04	55	5.18 (4.13 to 6.24)	0.48 (−0.37 to 1.34)	.27	−1.39 (−2.60 to −0.19)	.02
PCRS score^i^										
Baseline	60	119.62 (114.94 to 122.33)	NA	NA	60	118.63 (114.94 to 122.33)	NA	NA	NA	NA
4 mo	57	122.82 (118.90 to 126.74)	3.21 (0.80 to 5.61)	.009	55	118.71 (114.77 to 122.65)	0.08 (−2.36 to 2.51)	.95	3.13 (−0.29 to 6.55)	.07
12 mo	57	122.97 (118.57 to 127.37)	3.35 (0.46 to 6.25)	.02	54	120.21 (115.77 to 124.64)	1.57 (−1.38 to 4.52)	.30	1.78 (−2.35 to 5.91)	.40

Abbreviations: EQ-5D-5L, EuroQol 5-dimension 5-level; GAD-7, Generalized Anxiety Disorder 7-item scale; PART-O, Participation and Recombined Tools–Objective; PCRS, Patient Competency Rating Scale; PHQ-9, Patient Health Questionnaire 9-item scale; NA, not applicable; QOLIBRI, Quality of Life After Brain Injury; RPQ, Rivermead Post Concussion Symptoms Questionnaire.

^a^ 97.5% CI was used for primary outcomes and 95% CI for secondary outcomes.

^b^ Score range, 0 to 100, with higher scores indicating better health-related quality of life.

^c^ Score range, 0 to 5, with higher scores indicating higher levels of social participation.

^d^ Mean severity score of 3 main self-identified problems related to traumatic brain injury, which were individually assessed using a 4-point Likert scale, with 0 indicating not difficult at all and 4 indicating extremely difficult.

^e^ Score range, 0 to 1, with higher scores indicating better health-related quality of life.

^f^ Score range, 0 to 64, with higher scores indicating greater severity of symptoms.

^g^ Score range, 0 to 27, with higher scores indicating greater severity of depression.

^h^ Score range, 0 to 21, with higher scores indicating greater severity of anxiety.

^i^ Score range, 30 to 150, with higher scores indicating greater functional competency.

Within-group results indicated different trajectories in the groups. For the intervention group, statistically significant improvements were seen in all secondary outcomes (eg, target outcomes mean severity score at 12 months: −0.77; 95% CI, −1.01 to −0.53; *P* < .001; RPQ total score at 12 months: −7.03; 95% CI, −9.42 to −4.64; *P* < .001). The control group showed statistically significant improvements only in difficulty managing main TBI-related problems (target outcomes mean severity score at 12 months: −0.48; 95% CI, −0.72 to −0.24; *P* < .001) and TBI symptoms (RPQ total score at 12 months: −3.49; 95% CI, −5.91 to −1.07; *P* = .005) and a slight but statistically significant decrease in social participation (PART-O social subscale score at 12 months: −0.26; 97.5% CI, −0.44 to −0.07; *P* = .002) (Table 2).

## Discussion

This RCT aimed to evaluate the effect of a home-based, individually tailored, and goal-oriented intervention in improving HRQOL and participation, TBI-related difficulties, and symptoms. Although the 2 primary outcomes, disease-specific HRQOL (as measured by QOLIBRI overall scale scores) and social participation (as measured by PART-O social subscale scores), did not reveal between-group differences, we did see group effects in favor of the intervention group for the secondary outcomes of generic HRQOL, target outcomes severity, TBI-related symptoms, and anxiety.

The lack of significant between-group differences in the 2 primary outcomes warrants discussion. It is, however, noteworthy that a significant effect on HRQOL was found in the generic HRQOL measure. Some researchers argue that studies should include both generic and disease-specific measures of QOL.^31^ The decision to use the QOLIBRI overall scale to measure the primary outcome of HRQOL was based on the prevailing thought that disease-specific HRQOL measures might be more sensitive in revealing small but clinically meaningful differences.^32^ The QOLIBRI overall scale and the PART-O social subscale may both have been affected by COVID-19–related social restrictions. In addition, while the EQ-5D-5L has been widely used in research, the QOLIBRI overall scale was more recently developed, and its responsiveness in RCTs is unknown. The PART-O was chosen to measure the second primary outcome of participation because the scale is validated to measure participation after TBI. Due to the COVID-19 pandemic, only the social subscale of the PART-O was analyzed because it was thought to be less affected by the pandemic due its inclusion of digital types of socialization. However, a small decline in social participation was seen in both groups, but this decrease was statistically significant in the control group only. The reduced participation is likely a result of social distancing during the COVID-19 pandemic and might even have camouflaged a positive change in the intervention group because their social participation did not decline. In summary, although the lack of effect on primary outcomes may signify a lack of effect in targeted areas, it may also signify low sensitivity to change in the primary outcome measures.

This RCT was modeled after a study by Winter et al,^13^ which resulted in less difficulty with managing target outcomes and higher levels of community integration. Target outcomes are not well established in the field of rehabilitation, but they allow for an individualization of outcomes that might be particularly well suited to populations with heterogeneous difficulties, such as those with TBI.^15^ In this RCT, the severity of target outcomes differed between groups at 4 months only, which is comparable with findings from the study by Winter et al,^13^ which included only 1 outcome assessment. These effects were not maintained at 12 months. We can only speculate about the reasons for these temporal patterns. Decreases in intervention effects over time are well known to occur, as are study effects in control groups due to study procedures and follow-up.^33^ The findings highlight the need for development of plans to maintain rehabilitation intervention effects, such as prolonged follow-up and booster sessions.

Reductions in TBI- and anxiety-related symptoms were seen. Reduced anxiety may signify less worry and stress as participants experienced less difficulty managing TBI-related problems. The fact that the intervention group showed within-group improvements over time on all outcome measures except the PART-O social subscale supports this interpretation. High goal attainment has been previously documented in this trial,^34^ indicating positive intervention effects at an individual level. In a future trial, it would be of interest to include measures of self-efficacy because increased self-efficacy has been proposed to be a primary factor in transfer effects of rehabilitation interventions.^35^ The fact that intervention participants did not experience worsening in most of the outcomes during the follow-up period from 4 months to 12 months indicates sustainable effects over time.

This intervention aimed to address common health care needs among patients in the chronic phase of TBI. Although efforts were made to standardize intervention design and intensity, treatment content was individualized. There is an inherent challenge in measuring mean group effects of an intervention addressing individual needs, despite the fact that symptom heterogeneity requires individual tailoring of rehabilitation.^12^ This may partly explain the small differences between groups.

The fact that this intervention resulted in positive changes many years after injury confirms that rehabilitation in the chronic phase of TBI may be effective.^12^ Furthermore, the individually tailored nature of this intervention renders it a potential proof of principle study with relevance for patients with other chronic conditions, such as those with other neurological and brain injury etiologies. A similar approach to caregiver support has been shown to be effective in patients with dementia.^36^ The applicability of this approach in other populations should be evaluated in future studies.

### Limitations

This study has several limitations. It is important to emphasize that only participants with a radiologically verified intracranial injury were included. The eligibility criteria only allowed inclusion of patients with the capacity to contribute to goal setting and with self-reported TBI-related symptoms. Hence, results might not be applicable to patients with mild TBI without intracranial damage or patients with severe difficulties in awareness and cooperation in goal-setting procedures. Both primary outcomes may have been affected by the COVID-19 pandemic, but other findings nonetheless indicate effectiveness. Although this is one of the larger trials of individualized home-based rehabilitation in chronic TBI sequelae to date, the sample size is nonetheless limited. Both the sample size and the multiple outcomes applied in this trial entail a risk of both type 1 and type 2 errors. Lack of an active control group and exposure to follow-up in the control group (baseline interviews and reports to the participant’s general practitioner) could potentially bias results.

## Conclusions

In this RCT of a home-based, individually tailored, and goal-oriented intervention in the chronic phase of TBI, participants in the intervention group reported significantly improved generic HRQOL and fewer TBI- and anxiety-related symptoms, which were maintained at 12-month follow-up. These findings suggest that rehabilitation interventions could help patients in the chronic phase of TBI improve their HRQOL and symptom burden.

## Notes

Supplement 1.
Trial Protocol

Supplement 2. **eFigure.** Overview of Intervention Content

Supplement 3.
Data Sharing Statement
