# Supplementary material for: Effect of an Individually Tailored and Home-Based Intervention in the Chronic Phase of Traumatic Brain Injury: A Randomized Clinical Trial
Source: JAMA Netw Open. 2023 May 5;6(5):e2310821. doi: 10.1001/jamanetworkopen.2023.10821 (PMC10163390; doi:10.1001/jamanetworkopen.2023.10821)
Supplement: Supplement 1. — Trial Protocol [file jamanetwopen-e2310821-s001.pdf]

Protocol publication (including all changes): Borgen, I.M.H., Løvstad, M., Andelic, N. *et al.* Traumatic brain injury—needs and treatment options in the chronic phase: Study protocol for a randomized controlled community-based intervention. *Trials* 21, 294 (2020). https://doi.org/10.1186/s13063-020-4195-5

Protocol registered at Clinicaltrials.gov NCT03545594: Traumatic Brain Injury; Needs and Treatment Options in the Chronic Phase - Full Text View - ClinicalTrials.gov

Protocol change: Change was made to the original study protocol (below) based on experiences from the feasibility study. All changes were registered in Clinicaltrials before commencement.

**Change**	**Reason for change**
Family member participation optional	Feasibility (patients with rehabilitation needs, living far from their family)
Need for upper age limit	Feasibility (risk of neurodegenerative diseases increased
Abbreviation of neuropsychological protocol	Feasibility (baseline assessment found to be too long)
Change of SF-36 to QOLIBRI-OS/EQ-5D	Feasibility (assessments too long)
Addition of GAD-7	Feasibility (lacking assessment of anxiety-related symptoms)
Changed order of primary outcomes, QOLIBRI-OS and PART-O replaces Target Outcomes as co-primary outcomes	Feasibility (uncertainty regarding methodological quality of Target Outcomes, quality of life and participation considered important areas)
NPCS needs removed from outcomes	Lack of studies displaying its validity in the Norwegian population

Original protocol

Traumatic brain injury; needs and treatment options in the chronic phase. A randomized controlled community-based intervention.

Traumatic brain injury (TBI) is a condition affecting the persons' central nervous system with a need for extensive and highly specialized initial health care provision followed by comprehensive rehabilitation efforts. Frequent and life-long medical, cognitive, emotional and behavioral changes are expected. The community-based services are largely responsible for dealing with the chronic challenges affecting people with TBI.

The main study purpose and hypotheses

The main study purpose is to examine the long-term treatment needs and the effect of a goal-oriented community-based rehabilitation program for individuals with TBI living at home, with the following main research hypotheses:

- Person-centered intervention targeting the patients living environment will result in enhance goal achievement in participation and in lower burden of self-reported TBI related problems compared to the control group at the end of intervention, and 8 months after intervention (i.e., at 12 month after enrolling)
- Person-centered intervention will result in improved physical and mental health compared to the control group, at the end of intervention and 12 month follow-up.
- Person-centered intervention will result in fewer unmet health care needs compared to the control group, at the end of intervention and 12 month follow-up.
- Person-centered intervention will be a cost-effective alternative compared to the treatment as usual received by the control group.
- Patients, family members and rehabilitation professionals involved will be satisfied with the intervention program

Study design

Two-group RCT design.

Setting

In-home community based rehabilitation in the chronic phase of TBI (2-5 years post injury), in close collaboration with family members and local rehabilitation professionals.

Study population

- Patients (16-80 years) with clinical TBI diagnosis on acute admission and CT/MRI verified injury-related intracranial abnormalities at Oslo University Hospital (OUH)

- Patients reporting TBI-related cognitive, emotional, physical problems and/or reduced physical and mental health and/or difficulties with participation in activities with family, friends and in the community approximately 2 -5 years after the injury,

- Living at home at study inclusion, and having a family member or partner actively involved in his/her life.

Patients are excluded if they have severe preexisting neurological disorder or severe psychiatric diseases that would confound outcome assessments.

Recruitment and randomization

Eligible patients who consent to participation will be randomly allocated to either the intervention group or the control group in a 1:1 ratio. Internet-based block randomization with minimization will be used to ensure randomization and complete allocation concealment. Potential prognostic variables such as age, gender and injury severity will be balanced between groups. An investigator who is independent of the patient screening process will be responsible for allocating the patients to the study conditions. Blinding of the patients and rehabilitation professionals is not possible in this study, but the outcome assessors will be blinded to study group.

Group allocation and interventions

1. Pilot study: To help set-up the intervention programs and determine feasibility of the interventions, the recruitment process (participation, refusals) and the implementation of procedures and necessary adjustment regarding content and number of sessions, a pilot study involving 10 patients with their close family members (10% of total number) will be performed prior

to the full scale RCT. This pilot will also explore compliance from the patients, family members and local health professions, feasibly registrations of compliance as well as the time consumption and acceptability of the intervention.

2. *The patient-centered in-home program intervention* will be modeled with reference to the Winter et al. study (1), which consists of eight contacts (six in-home visits of 1-2 hours duration each and two telephone contacts), delivered over a 4-month period in three phases: Phase I: assessment of patients' needs and main activity limitations (target outcomes) in collaboration with the patient, family member and patient family doctor or responsible local rehabilitation health care professional, along with review of the purpose of intervention. Phase II: Defining specific goals and action planning based on the SMART goals model, where SMART goals need to be Specific, Measurable, Achievable, Realistic, and Timed (2). This ensures an individual goal setting plan documenting targeted activities and strategies for each identified problem in multi-level domains of functioning (contacts 1-3); Phase III: establishment of strategies to modify the physical and psycho-social environment and of compensatory strategies and training to deal with targeted symptoms, included a final in-home visit (contact 3-8).

Establishment of functional target outcome areas and SMART-goals

Identifying target outcome areas as experienced by both the patients and their relatives, is done by asking them to list the most and the next most troubling symptom due to their brain injury, as well as indicate on a 5-point Likert scale how difficult these problems are to deal with (from 0=not at all, to 4=extremely).

Up to three target outcomes will be identified. The Likert scale evaluation of the target outcomes will also be used as an outcome measure. There might be marked discrepancies in patients´ and relatives' subjective experience of symptoms and how troubling they are. Therefore, specific SMART goals will be established (2), based on the target outcomes, where both the perspective of the patient and the family member will be taken into account. Goals will be ensured to address functional domains in every day living. There might thus be more than one specific goal connected to each target outcome area. Goal attainment will be measured at outcome using Goal Attainment Scaling (GAS) (3). Goals are classified according to their importance, on a scale of 0–3 ranging from 0="not at all" to 3="very" important. Goal attainment is classified as being between +2 and -2, where a score of 0 represents goal attainment at the expected level pre-treatment, -1 and -2 correspond to a little and a lot worse goal attainment than expected, respectively, and likewise, +1 and +2 represent goal attainment that is a little more or a lot more than anticipated.

This represents a highly structured way of establishing the functional areas that patients and relatives want to address, along with establishment of specific treatment goals, and ensures that the treatment relates to personally relevant problem areas. The Likert scale evaluation of severity of target outcomes, along with scoring of goal importance and goal attainment, additionally ensures that a patient-centered perspective can be combined with quantifiable outcomes that are comparable across participants.

Intervention content

Based on well-established knowledge about the most common long-term symptoms after moderate to severe TBI, and the experiences of Winter et al (1;4;5), we expect that target outcomes and related goals will predominantly be related to cognitive (e.g. memory, attention, executive functioning & self-awareness, communication), physical (e.g. sensory and motor deficit, fatigue, dizziness, reduced balance, visual problems), emotional (e.g. anxiety and depression secondary to injury, stress management), and interpersonal problems (e.g. reduced awareness of deficit, personality changes, disinhibited behavior, apathy, irritability).

In addition to providing a structured treatment program, one main goal of this study is for the specialist health care system to contribute to community-based rehabilitation being in accord with evidence based practice. In order to ensure high quality interventions, the study will therefore include components from evidence-based treatment programs within relevant functional domains, enabling adaptation of specialized rehabilitation programs to the home-setting. Interventions addressing cognition will be in accord with state-of-the art reviews of cognitive rehabilitation (6). The *Translating Evidence-Based Recommendations into Practice document* will also inform intervention content. Choice of interventions will adhere to the recommended decision trees in aforementioned reviews. As the current study involves TBI with intracranial injuries revealed at the neuroimaging in the acute phase, while Winter and colleagues predominantly treated mild injuries, symptom severity is expected to be of more severe grade. An important principle within TBI rehabilitation is to start out with establishing symptom severity, along with the patients´ symptom awareness, and consequently, motivation for rehabilitation efforts targeting that specific domain. In general, the more severe the symptoms are, the more the interventions need to

involve environmental modifications. On the other hand, in cases of mild to moderate symptom burden, and good symptom awareness, the interventions can to a larger degree be based on training of internal compensatory strategies and metacognitive strategies. Systematic use of compensatory devices and external aids will be part of the intervention tool-box.

Muscle relaxation and mindfulness techniques will be used to address problems with stress management. Regarding symptoms of anxiety and depression, techniques derived from Cognitive Behavioral Therapy will be the dominating theoretical approach, although an eclectic stance will be taken, e.g. in cases where threats to identity and self-concept are seen to be more readily addressed using other therapeutic approaches (7).

Based on the identified target outcomes and the corresponding SMART-goals, an action plan will be established for each patient, in accord with Winter et al. In the action plan, the specific interventions chosen in relation to each goal will be described. The flow-chart below describes the process of establishing a specified and patient-centered intervention strategy with corresponding measurable outcomes.

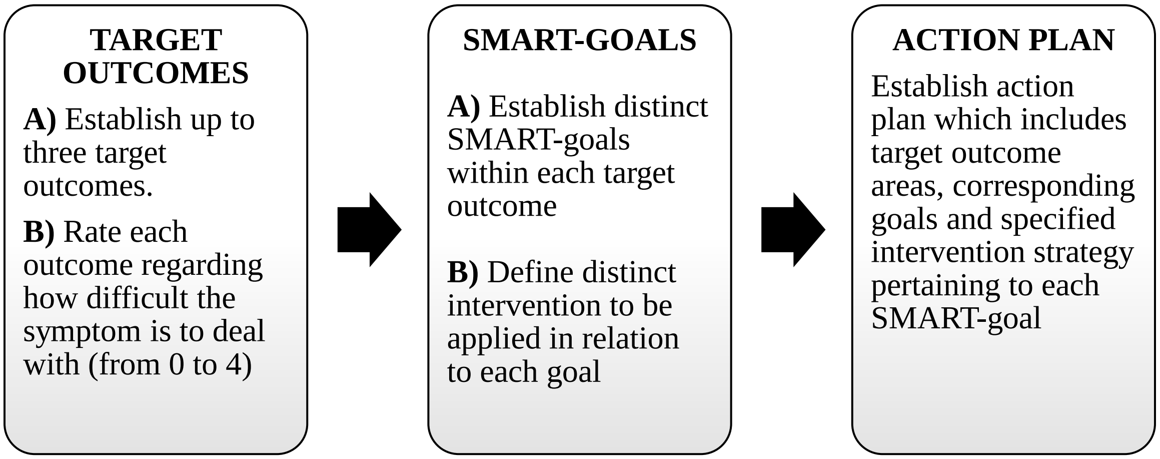

Received services during the study period will be registered by the Needs and provision complexity scale part B.

Procedure – collaboration with the local health care professionals and intervention delivery: the patient will nominate his/her primary local health care professional to join the intervention sessions. For participants who are not connected to a local health care professional, the study investigators will establish such a contact in collaboration with the patient´s general medical doctor. The involvement of the municipal health care professional in the intervention will ensure lasting knowledge transference. The principal investigator in collaboration with senior TBI researchers will supervise the study interventionists (trained allied neurorehabilitation professionals). Concerns about the protocols will be discussed and resolved in project meetings throughout the project period.

3. *The control group* will receive the usual follow-up assessment and health care and rehabilitation services provided in the municipality. At the time of inclusion, patients in the control group will self-identify their main problems and rehabilitation goals for the next 4-6 months. The received services will be registered and mapped according to the ICSO-R over the study period. The control group will be followed-up by two attention-telephone calls to maintain contact with participants and to registered any changes that might have occurred and reminders about the follow-up interviews

Data collection

Baseline assessment of cognitive, emotional, functional and participation status will be performed at inclusion (= T1), with outcome assessments being performed immediately following the intervention (= T2) and 8 months after end of the intervention (= T3).

The assessments will be performed at the outpatient clinic of Department of Physical Medicine and Rehabilitation,Oslo University Hospital.

Patient characteristics

The following socio-demographic variables will be recorded at baseline: age, gender, marital status, living conditions, educational level and description of pre-injury employment. Medical variables including comorbidity, injury characteristics and clinical severity (Glasgow Coma Scale Score, Posttraumatic amnesia), neuroimaging results, length of acute and inpatients hospitalizations, medical and rehabilitation treatment modalities during the first year after the injury will be derived from the medical journal. In addition, the data regarding participation in paid employment or education and the use of health care and rehabilitation services beyond the first year of injury will be collected. Assessment of patients' needs for health care and social support services by patient, family member and clinician will also be collected at the study inclusion time, along with a neuropsychological screening of IQ, memory, processing speed and executive functioning (Vocabulary, Similarities, Block design and Matrix Reasoning from the Wechsler Adult Intelligence Scale (WAIS-IV), California Verbal Learning Test-II, the Processing Index from the Wechsler Adult Intelligence Scale–IV, the Trail Making and Color Word Interference Tests from the

Delis-Kaplan Executive Function System, Behavior Rating Inventory of Executive Function Adult Version (BRIEF-A).

Outcome assessment

Severity of target outcomes, goal attainment, participation in the community and met health care needs are the main areas targeted, and will be focused in the assessments and outcome evaluations along with cognitive, emotional and global functioning measures, and satisfaction with services (Table 1), based on the TBI common data elements and Winter et al (1;8).

Table 1. Description of the outcome measures and domains targeted.

All assessments will be applied at baseline, after the intervention and at 12 month follow-up (8 months after the intervention)

**Assessments and outcome measures**	**General description of targeted domains**
**<u>Primary outcome measures</u>**	
- Target outcomes and their severity	Individually identified target functional domains and their severity
- **Goal Attainment Scaling** (GAS)	Goal achievement
- **Needs and provision complexity scale** (Part A NPCS needs; Part B: NPSC- gets)	Needs for rehabilitation and social support

<u>**Secondary outcomes measures**</u><br><br>**- Participation assessment and Recombined Tools- Objective** (PART-O)	Participation
**- Glasgow outcome scale extended** (GOSE)	Global outcome
**Rivermead post-concussion questionnaire** (RPQ)	Symptom burden
**Patients Health Questionnaire** (PHQ-9)	Depression
**SF-36/ SF-6D**	Physical and mental health and quality-adjusted life years, (QALYs)
**Patient Competency Rating Scale (PCRS)**	Patient and family members, evaluating self-awareness and functioning.
**Acceptability Scale (18 items)**	Acceptability of intervention<br><br>assessed by patient, family member and health professional

Parameters for process evaluation

The participation rate, numbers of consultations, the direct and indirect time related to each consultation, the kind of problems presented, completion of intervention due to protocol and in case of non-compliance, reason for non-compliance. After completion of the intervention, the participants and family members will be asked to evaluate the intervention (9) and how satisfied they are with the intervention.

Determination of costs

Data concerning cost will be gathered prospectively using monthly cost-diaries. All participants will be asked to note all their health care utilizations during that month. For the calculation of total costs, direct health care costs, direct non-health costs and indirect costs will be recorded such as visits to health care providers and medication costs, the costs of informal health care, cost of health activities, hours of paid or unpaid household help and special aids as well as loss of paid and unpaid work productivity of the patient. Costs of interventions will also be calculated.

Analysis and statistics

All data will be anonymously coded and registered electronically in SPSS. T-test analysis will be used for between-group mean comparisons for normally distributed continuous data, and Mann Whitney U-tests for skewed data. Regression analysis will be applied using the main variables of interest to predict outcomes. In order to test whether the intervention has a beneficial effect on goal attainment, participation symptom level and functioning, compared to the control group, mixed model analysis will be used with time (T1-T3) as the repeated-measures factor, and comparison between the intervention and control groups being a between group factor. Intention to treat analyses will be performed adjusted for sociodemographic and service content variables from ICSO-R. To evaluate the effect of the intervention, 50 patients need to be completing the intervention in each group, based on the experiences from Winter et al (1), which required power of 90% and a significance level of 0,05. With an estimated loss to follow-up of 20%, based on our earlier TBI research, 120 participants are thought to be necessary and sufficient for this trial. Intention to treat analyses will be performed for the randomized trial. The descriptive approach will be used for analyses of process evaluation.

To determine the cost-effectiveness of the intervention, a statistical analysis of costs will be performed. The total costs will be calculated by adding up direct health care costs, direct non-health costs and indirect costs. As the distribution of costs can be skewed, differences in costs between groups will be calculated by means of bootstrapping. A cost-utility analysis will relate the difference between the intervention and control group to changes in utility. This will result in costs per quality-adjusted-life years (QALYs). QALYs can be derived from the SF-36 data using SF-6D (10) Standard discounting will be performed for both costs and outcomes together with sensitivity and uncertainty analysis to gain insight into the generalizability of the economic evaluation.

Reference List

(1) Winter L, Moriarty HJ, Robinson K, Piersol CV, Vause-Earland T, Newhart B, et al. Efficacy and acceptability of a home-based, family-inclusive intervention for veterans with TBI: A randomized controlled trial. Brain Inj 2016 Mar 16;1-15.

(2) Bovend'Eerdt TJ, Botell RE, Wade DT. Writing SMART rehabilitation goals and achieving goal attainment scaling: a practical guide. Clin Rehabil 2009 Apr;23(4):352-61.

(3) Malec JF. Goal attainment scaling in rehabilitation. Neuropsychological rehabilitation 1999;9(3/4):253-75.

(4) Anke A, Andelic N, Skandsen T, Knoph R, Ader T, Manskow U, et al. Functional Recovery and Life Satisfaction in the First Year After Severe Traumatic Brain Injury: A Prospective Multicenter Study of a Norwegian National Cohort. J Head Trauma Rehabil 2014 Jul 16.

(5) Andelic N, Soberg HL, Berntsen S, Sigurdardottir S, Roe C. Self-Perceived Health Care Needs and Delivery of Health Care Services 5 Years After Moderate-to-Severe Traumatic Brain Injury. PM R 2014 May 15.

(6) Cicerone KD, Langenbahn DM, Braden C, Malec JF, Kalmar K, Fraas M, et al. Evidence-based cognitive rehabilitation: updated review of the literature from 2003 through 2008. Arch Phys Med Rehabil 2011 Apr;92(4):519-30.

(7) Pastore V, Colombo K, Liscio M, Galbiati S, Adduci A, Villa F, et al. Efficacy of cognitive behavioural therapy for children and adolescents with traumatic brain injury. Disabil Rehabil 2011;33(8):675-83.

(8) Wilde EA, Whiteneck GG, Bogner J, Bushnik T, Cifu DX, Dikmen S, et al. Recommendations for the use of common outcome measures in traumatic brain injury research. Arch Phys Med Rehabil 2010 Nov;91(11):1650-60.

(9) Gitlin LN, Hodgson N, Jutkowitz E, Pizzi L. The cost-effectiveness of a nonpharmacologic intervention for individuals with dementia and family caregivers: the tailored activity program. Am J Geriatr Psychiatry 2010 Jun;18(6):510-9.

(10) Brazier J. Measuring and valuing mental health for use in economic evaluation. J Health Serv Res Policy 2008 Oct;13 Suppl 3:70-5.
