# Supplementary material for: Effect of an Individually Tailored and Home-Based Intervention in the Chronic Phase of Traumatic Brain Injury: A Randomized Clinical Trial
Source: JAMA Netw Open. 2023 May 5;6(5):e2310821. doi: 10.1001/jamanetworkopen.2023.10821 (PMC10163390; doi:10.1001/jamanetworkopen.2023.10821)
Supplement: Supplement 2. — eFigure. Overview of Intervention Content [file jamanetwopen-e2310821-s002.pdf]

Supplementary Online Content

Borgen IMH, Løvstad M, Hauger SL, et al. Effect of an individually tailored and home-based intervention in the chronic phase of traumatic brain injury: a randomized clinical trial. *JAMA Netw Open*. 2023;6(5):e2310821. doi:10.1001/jamanetworkopen.2023.10821

eFigure. Overview of Intervention Content

This supplemental material has been provided by the authors to give readers additional information about their work.

© 2023 Borgen IMH et al. *JAMA Network Open*.

eFigure. Overview of Intervention Content

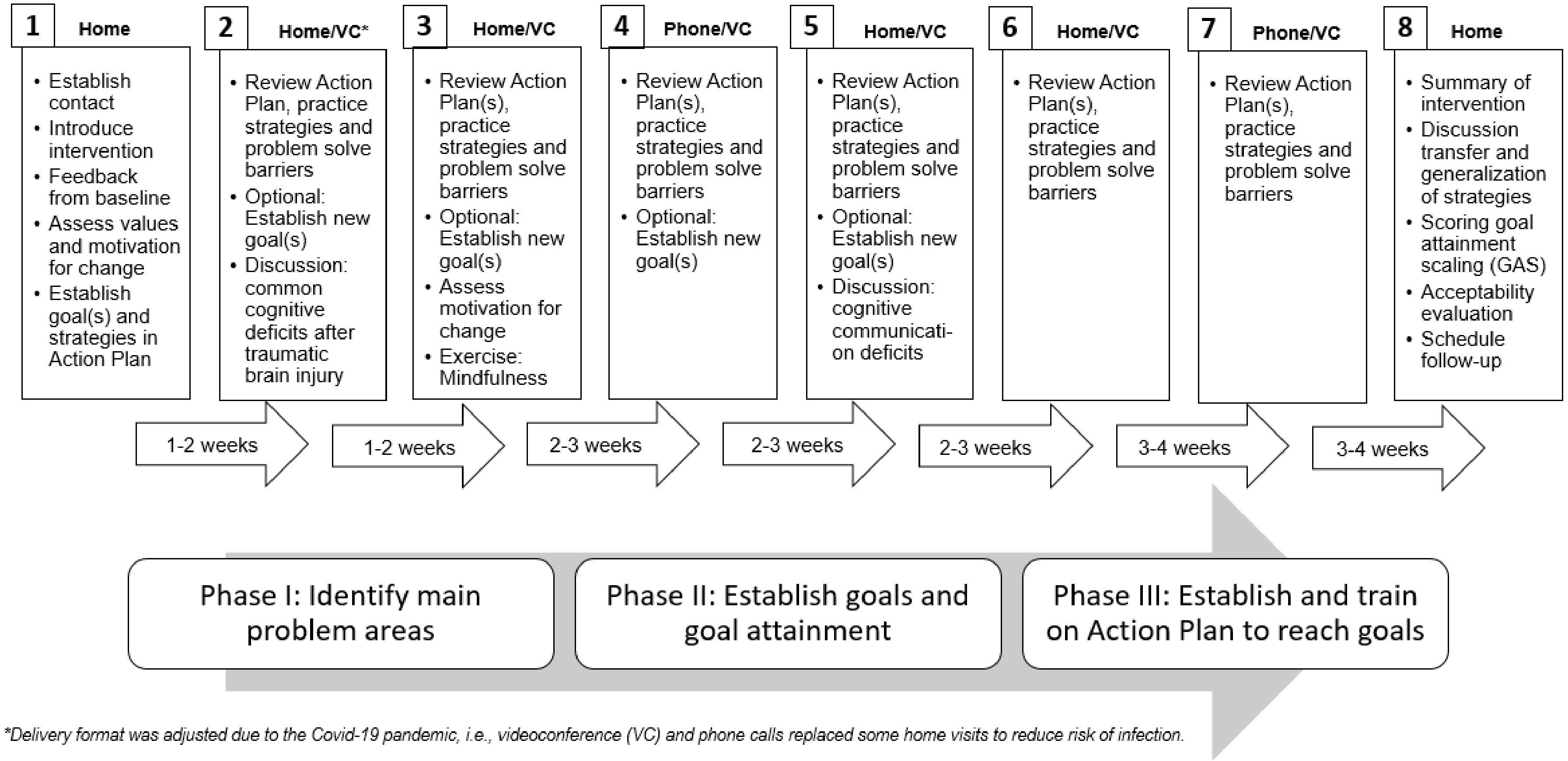

Delivery format was adjusted due to the Covid-19 pandemic, i.e., videoconference (VC) and phone calls replaced some home visits to reduce risk of infection.

© 2023 Borgen IMH et al. *JAMA Network Open*.
