# Supplementary material for: Effect of an Individually Tailored and Home-Based Intervention in the Chronic Phase of Traumatic Brain Injury: A Randomized Clinical Trial
Source: JAMA Netw Open. 2023 May 5;6(5):e2310821. doi: 10.1001/jamanetworkopen.2023.10821 (PMC10163390; doi:10.1001/jamanetworkopen.2023.10821)
Supplement: Supplement 3. — Data Sharing Statement [file jamanetwopen-e2310821-s003.pdf]

Data Sharing Statement

Borgen. Effect of an Individually Tailored and Home-Based Intervention in the Chronic Phase of Traumatic Brain Injury. *JAMA Netw Open*. Published May 05, 2023.
doi:10.1001/jamanetworkopen.2023.10821

Data
Data available: No

Additional Information
Explanation for why data not available: In accordance with Norwegian Data Protection Laws, the data set will not be made available to the public. Anyone wishing to view the originial data can do so by request to the corresponding author and physical presence at Oslo University Hospital, Norway.
